# Value of preoperative ureteral wall thickness in prediction of impaction of ureteric stones stratified by size in laser ureteroscopic lithotripsy

**DOI:** 10.1186/s12894-022-01168-4

**Published:** 2023-01-06

**Authors:** Abdrabuh M. Abdrabuh, El-Sayed I. El-Agamy, Mohamed A. Elhelaly, Tamer A. Abouelgreed, Ibrahim Abdel-Al, Hamada A. Youssof, Adel Elatreisy, Osama Shalkamy, Mohamed Elebiary, Mohammed Agha, Ibrahim Tagreda, Ahmed Alrefaey, Elsayed Elawadey

**Affiliations:** 1 Urology Department, Al-Azhar University, Cairo, Egypt; 2 Urology Department, Al-Azhar University, Assuit Branch, Assuit, Egypt; 3 Urology Department, Faiyum University, Faiyum, Egypt; 4 Present Address: Armed forced Hospital, Alhada, Saudi Arabia

**Keywords:** Ureteric stones, Ureteral wall thickness, Impaction, Laser

## Abstract

**Objectives:**

To evaluate the role of preoperative UWT in the prediction of impaction of ureteral stones stratified according to stone size in ureteroscopic laser lithotripsy.

**Patient and methods:**

This study included 154 patients submitted to URSL for ureteral stones. Radiological data comprised the presence of hydronephrosis, anteroposterior pelvic diameter (PAPD), proximal ureteric diameter (PUD), and maximum UWT at the stone site. Collected stone characteristics were stone size, side, number, site, and density.

**Results:**

The study included 154 patients subjected to URSL. They comprised 74 patients (48.1%) with impacted stones and 80 (51.9%) with non-impacted stones. Patients were stratified into those with stone size ≤ 10 mm and others with stone size > 10 mm. In the former group, we found that stone impaction was significantly associated with higher PAPD, PUD, and UWT. In patients with stone size > 10 mm, stone impaction was related to higher UWT, more stone number, and higher frequency of stones located in the lower ureter. ROC curve analysis revealed good power of UWT in discrimination of stone impaction in all patients [AUC (95% CI) 0.65 (0.55–0.74)] at a cut-off of 3.8 mm, in patients with stone size ≤ 10 mm [AUC (95% CI) 0.76 (0.61–0.91)] at a cut-off of 4.1 mm and in patients with stone size > 10 mm [AUC (95% CI) 0.72 (0.62–0.83)] at a cut-off of 3.0 mm.

**Conclusions:**

Stratifying ureteric stones according to size would render UWT a more practical and clinically-oriented approach for the preoperative prediction of stone impaction.

## Background

Ureteral stone impaction is a common encounter that entails many clinical and operative risks and challenges. Long-standing ureteral stone impaction is frequently complicated by ureteral polyps and strictures [1–3]. Also, it was found that stone impaction is related to a higher risk of associated sepsis [4, 5]. Moreover, it was found that impacted stones are predictive of lower stone-free rates after ureterolithotripsy [6]. In addition, the rate of major ureteroscopic complications was found to be increased with stone impaction [7, 8]. Moreover, it was noted that stone impaction negatively affects the success rate of laser endoureterotomy of ureteral strictures [9].

Considering the described drawbacks of ureteric stone impaction, it would be useful to predict stone impaction preoperatively. However, the prediction of ureteral stone impaction is a challenging issue. This is attributed to the multiplicity of factors implicated in the pathogenesis of the impaction process [10]. In this context, many predictive factors were suggested. Examples include higher ureteral density on preoperative CT [11], degree of hydronephrosis, and ureteral wall thickness (UWT) [12–15]. In their work on the clinical significance of UWT in the prediction of stone impaction in patients subjected to ureteroscopic lithotripsy, Yoshida et al. [12] suggested a UWT of 3.49 mm as an optimal cut-off for the prediction of impacted stones.

The present study argues that adopting a particular cut-off value, regardless of other factors contributing to stone impaction, particularly stone size needs more justification. In this work, we evaluated the role of preoperative UWT in the prediction of impaction of ureteral stones stratified according to stone size during URSL.

## Patients and methods

The present prospective study was conducted at urology department in our tertiary care hospital, from August 2018 to April 2021. The local ethical committee approved the study protocol. The informed consent was obtained from all subjects and/or their legal guardian(s). The study included 154 patients who submitted to consecutive URSL procedures for ureteral stones after failed medical explosive therapy consisting of 0.4 mg daily night-time tamsulosin and oral three times daily k citrate. Eleven patients were excluded because preoperative NCCT was not performed, and 43 underwent URSL only for renal stones. For all patients, we reported clinical data, including age, sex, BMI, history of the previous URSL, duration of symptoms, and Eastern Cooperative Oncology Group (ECOG) performance status. Radiological data were collected using a low threshold CT scan which comprised the presence of hydronephrosis, pelvic anteroposterior diameter (PAPD), proximal ureteric diameter (PUD), and maximum UWT at the stone site. All measurement was done in the direct axial image using a Philips machine. The thickness of the slices was measured every 2.5 mm for each cut. One expert radiologist did all measurements. Reported stone characteristics were stone size, side, number, site, and density. In our study, stone impaction was defined as staying at the same position in the ureter for at least two months and failing to pass a guide wire through the stone at the initial attempt [1]. All URLS procedures were conducted according to the standard surgical protocols using a 6.4/7.8- Fr semi-rigid ureteroscope (Olympus, Tokyo, Japan) for lower and middle ureteric stones and 7.95-Fr flexible Ureteroscopy URF-P6 (Olympus) and holmium laser lithotripsy (Lumenis PluseTM 100H Germany) for upper ureteric stones. The setting of the holmium laser to disintegrate the stone used an energy level of 0.8–1.2 J and a rate of 5–10 Hz. The total energy delivered by laser was fixed for all patients, and no changes in total energy between groups. A standard guide wire (0.038 Ch) for all patients and a ureteric catheter 6 Fr were used to support the wire during the introduction. In most cases, a ureteral access sheath (9.5/11.5 Fr) was placed. If possible, a tipless nitinol basket (1.5–1.7F) was used to extract the stone fragments. An indwelling ureteric stent was inserted in all patients. All procedures were done by a single expert surgeon.

The study's primary outcome was the successful ureteroscopic LASER lithotripsy estimating the difference between the study groups regarding the endoscopic picture of the ureteric wall, Lasering time, and operative time.

### Statistical methods

The collected data were organized, tabulated, and statistically analyzed using a statistical software package for social science (SPSS) version 25 (SPSS Inc, USA). The included sample size was sufficient to provide the effect of ureteral wall thickness in the Prediction of impaction of Ureteric Stones with a power of 80% and an alpha error of 0.05. To calculate the sample size, which means the minimum number of necessary samples to meet the desired statistical constraints Using the Minitab program (Version 17), we supposed the proportion incidence of cases under study is 1% and the Confidence level is 95% with Confidence interval upper bound; moreover, the sample size is 154 cases with 0.05 margins of error.

Descriptive statistics were performed for all study variables with a normality test for all quantitative variables. Data were explored for normality using the Kolmogorov–Smirnov test and Shapiro–Wilk test. Numerical data were summarized using means and standard deviations or medians and ranges. Categorical data were summarized as (numbers) percentages. Comparisons between the two groups concerning normally distributed numeric variables were made using the independent t-test or Mann–Whitney test for non-normally distributed numeric variables. For categorical variables, differences were analyzed with χ2 (chi-square). Pearson’s correlation coefficient was used to perform correlation analysis. Receiver operator characteristic (ROC) curve analysis was used to determine cut-offs, sensitivity, and specificity of UWT to identify impacted stones. Logistic regression analysis was used to identify predictors of stone impaction. P value less than 0.05 was considered statistically significant. All statistical operations were processed using SPSS 25 (IBM, USA) [16].

## Results

The present study included 154 patients subjected to URSL. They comprised 74 patients (48.1%) with impacted stones and 80 patients (51.9%) with non-impacted stones. The clinical and radiological characteristics of the studied patients are shown in Table 1. Comparison between patients with impacted stones and patients without impacted stone revealed that patients with impacted stones are significantly older (*p* value = 0.05), with significantly higher PUD (*p* value < 0.001) and UWT (*p* value < 0.001) and larger stone size (*p* < 0.001) and more frequency of stones located in the lower ureter (*p* value = 0.03) (Table 1).

**Table 1:** Comparison between patients with stone impaction and patients without according to stone size regarding the clinical and outcome parameters

	All patients N = 154	Impacted n = 74	Non impacted n = 80	*p*
Age (years) mean ± SD	37.0 ± 8.9	38.7 ± 8.1	36.5 ± 8.4	0.05
Male/female n	86/48	39/35	47/33	0.24
BMI (kg/m^2^)	29.5 ± 3.5	29.5 ± 3.5	29.4 ± 3.3	0.91
Previous URSL n (%)	47 (30.5)	23 (31.1)	24 (30)	0.82
Duration of symptoms (days) median (range)	29 (13.0–152.0)	32 (13 -152)	21 (13 -148)	0.06
Duration of medical treatment prior to URS (days)	47.9 ± 9.4	50.8 ± 10.5	45.2 ± 7.5	< 0.001
*ECOG performance status n (%)*				
< 2	125 (81.2)	57 (77)	68 (85)	0.1
≥ 2	29 (19.8)	17 (23)	12 (15)	
*Radiological findings*				
Hydronephrosis n (%)	134 (87.0)	69 (93.2)	65 (81.3)	0.05
Hydronephrosis grade n (%)				
Mild	108 (70.1)	49 (66.2)	59 (73.8)	0.04
Moderate	26 (16.9)	20 (27)	6 (7.5)	
PAPD (mm) mean ± SD	25.2 ± 7.5	27.2 ± 9.3	23.5 ± 6.9	< 0.001
PUD (mm) mean ± SD	11.3 ± 3.8	13.2 ± 3.7	10.1 ± 3.6	< 0.001
UWT (mm) mean ± SD	3.1 ± 1.7	3.91 ± 1.9	2.72 ± 1.68	< 0.001
*Stone characteristics*				
Size (mm) mean ± SD	12.0 ± 3.4	14.2 ± 2.9	10.3 ± 3.4	< 0.001
Side (right/left) n	82/72	35/39	47/33	0.28
[Number (n) mean ± SD	1.1 ± 0.4	1.24 ± 0.5	1.07 ± 0.3	0.03
Site n (%)				
Upper	58 (37.7)	30 (40.5)	28 (35)	0.03
Middle	24 (15.6)	3 (4.1)	21 (26.2)	
Lower	72 (46.7)	41 (55.4)	31 (38.8)	
Density (HU)	470.9 ± 163.6	468.1 ± 167.4	469.8 ± 162.9	0.82
*Endoscopic findings n (%)*				
Ureteral edema	114 (74.0)	50 (67.6)	64 (80)	0.1
Ureteral polyp	33 (21.4)	23 (31.1)	10 (12.5)	
White patch	7 (4.6)	1 (1.3)	6 (7.5)	

*BMI* body mass index, *ECOG* Eastern Cooperative Oncology Group, *PAPD* pelvic anteroposterior diameter, *PUD* proximal ureteric diameter, *URSL* ureteroscopic lithotripsy, *UWT* ureteral wall thickness

Our study further stratified included patients into those with stone size ≤ 10 mm and others with stone size > 10 mm. In the former group, we found that stone impaction was significantly associated with higher PAPD (*p* value = 0.08), PUD (*p* value = 0.04), UWT (*p* value = 0.005), operative time (*p* value = 0.03), and lasering time (*p* value = 0.04). In comparison, in patients with stone size > 10 mm, stone impaction was related to higher UWT (*p* value < 0.001), more stone number (*p* value = 0.01), operative time (*p* value = 0.04), lasering time (*p* value 0.03) and higher frequency of stones located in the lower ureter (*p* value = 0.01) (Table 2). In our study no postponement of treatment or conversion to open or laparoscopic surgery.

**Table 2:** Comparison between patients with stone impaction and patients without stone impaction stratified according to stone size regarding the clinical and outcome parameters

	Size ≤ 10 mm n = 60		*p*	Size > 10 mm n = 94		*p*
	Impacted n = 16	Not impacted n = 44		Impacted n = 58	Not impacted n = 36	
Age (years) mean ± SD	33.9 ± 3.1	34.1 ± 7.2	0.93	40.4 ± 9.2	37.5 ± 9.4	0.1
Male/female n	7/9	27/17	0.33	32/26	20/16	0.69
BMI (kg/m^2^)	29.7 ± 3.6	29.5 ± 3.7	0.78	29.8 ± 3.1	29.7 ± 3.6	0.81
Previous URSL n (%)	4 (25)	12 (26.1)	0.8	21 (36.2)	10 (27.8)	0.31
Duration of symptoms (days) median (range)	38 (13.0–146.0)	21 (16.0–145.0)	0.21	29.0 (13.0–152.0)	24.0 (13.0–148.0)	0.11
*ECOG performance status n (%)*						
< 2	10 (62.5)	37 (84.1)	0.21	48 (82.8)	30 (83.3)	0.44
≥ 2	6 (37.5)	7 (15.9)		10 (17.2)	6 (16.7)	
*Radiological findings*						
Hydronephrosis n (%)	13 (81.3)	36 (81.8)	0.93	56 (95.6)	29 (80.6)	0.04
Hydronephrosis grade n (%)						
Mild	9 (56.3)	36 (81.8)	0.05	40 (68.9)	23 (63.9)	0.09
Moderate	4 (25)	1 (2.3)		16 (27.6)	5 (13.9)	
PAPD (mm) mean ± SD	27.9 ± 10	23.4 ± 5.2	0.08	28.6 ± 8.2	25.1 ± 6.3	0.09
PUD (mm) mean ± SD	11.9 ± 2.8	9.6 ± 3.9	0.04	13.2 ± 3.4	11.1 ± 4.1	0.03
UWT (mm) mean ± SD	5 ± 1.6	3.6 ± 1.4	0.005	3.7 ± 1.6	2 ± 1.4	< 0.001
*Stone characteristics*						
Size (mm) mean ± SD	8.9 ± 1.4	8.5 ± 1.3	0.64	14.7 ± 2.2	13.5 ± 3.7	0.47
Side (right/left) n	9/6	25/14	0.23	26/33	22/19	0.1
Number (n) mean ± SD	1.1 ± 0.1	1 ± 0.4	0.31	1.33 ± 0.7	1.0 ± 0.3	0.01
Site n (%)						
Upper	5 (31.3)	13 (29.6)	0.2	25 (43.1)	15 (41.7)	0.01
Middle	1 (6.3)	10 (22.7)		2 (3.4)	11 (20.6)	
Lower	11 (68.8)	21 (47.7)		30 (71.4)	10 (27.8)	
Density (HU)	368.7 ± 132.6	409.5 ± 129.1	0.17	499.7 ± 179.5	501 ± 177	0.93
*Endoscopic findings n (%)*						
Ureteral edema	8 (50)	39 (88.6)	0.1	42 (72.4)	25 (69.4)	0.21
Ureteral polyp	2 (12.5)	2 (4.6)		21 (36.2)	8 (22.2)	
White patch	-	-		1 (2.4)	6 (11.5)	
Operative Time(min) mean ± SD	46 ± 1.4	27.9 ± 1.3	0.03	55 ± 1.2	35 ± 1.6	0.04
Lasering time(min) mean ± SD	27.2 ± 2	13.9 ± 1.1	0.04	33 ± 1.6	17 ± 1.8	0.03

*BMI* body mass index, *ECOG* Eastern Cooperative Oncology Group, *PAPD* pelvic anteroposterior diameter, *PUD* proximal ureteric diameter, *URSL* ureteroscopic lithotripsy, *UWT* ureteral wall thickness, *HU* hounsfield unit

Correlation analysis revealed significant direct correlation between UWT and PAPD (*p* value = 0.025), PUD (*p* value < 0.001) and stone density (*p* value = 0.002) in the whole cohort. In patients with stones ≤ 10 mm, UWT was significantly correlated with PAPD (*p* value < 0.001) and PUD (*p* value < 0.001) while in those with stones > 10 mm, UWT was correlated with PUD (*p* value < 0.001) and stone density (*p* value = 0.02) (Table 3).

**Table 3:** Correlation between UWT and clinical and radiological data

	All patients		Patients with stone ≤ 10 mm		Patients with stone > 10 mm	
	r	p	r	p	r	p
Age	0.04	0.67	0.13	0.34	0.1	0.35
BMI	− 0.14	0.1	− 0.12	0.38	− 0.13	0.23
Duration of symptoms	0.083	0.31	− 0.021	0.87	0.066	0.53
PAPD	0.18	0.025	0.54	< 0.001	0.09	0.4
PUD	0.28	< 0.001	0.44	< 0.001	0.37	< 0.001
Stone size	− 0.16	0.051	0.23	0.08	0.05	0.61
Stone number	0.03	0.72	− 0.1	0.47	0.11	0.31
Stone density	− 0.25	0.002	− 0.04	0.75	− 0.24	0.021

*BMI* body mass index, *PAPD* pelvic anteroposterior diameter, *PUD* proximal ureteric diameter

In multivariate analysis, logistic regression analysis identified UWT (*p* value < 0.001), stone size (*p* value < 0.001) and stone location in the middle and lower ureter (*p* value = 0.005) as predictors of stone impaction in all patients. In patients with stone size ≤ 10 mm, only UWT (*p* value = 0.01) could predict stone impaction. In patients with stone size > 10 mm, predictors of stone impaction included UWT (*p* value < 0.001) and stone location in the lower ureter (*p* value = 0.002) (Table 4).

**Table 4:** Predictors of stone impaction in patients groups

	Univariate analysis			Multivariate analysis		
	OR	95% CI	*p*	OR	95% CI	*p*
*All patients*						
Age	1.05	1.0–1.09	0.02	1.04	0.99–1.09	0.15
Hydronephrosis	3.06	0.85–11.0	0.086	–	–	–
UWT	1.44	1.16–1.79	0.001	2.23	1.58–3.13	< 0.001
Stone size	1.17	1.05–1.31	0.004	1.36	1.18–1.56	< 0.001
Stone site						
Upper	Ref					
Middle	0.17	0.04–0.81	0.026	0.08	0.01–0.5	0.007
Lower	1.21	0.59–2.48	0.61	4.37	1.57–12.2	0.005
*Patients with stone size ≤ 10 mm*						
Age	1.0	0.91–1.12	0.93	–	–	–
Hydronephrosis	0.79	0.09–7.28	0.83	–	–	–
UWT	2.07	1.16–3.69	0.014	2.07	1.16–3.69	0.014
Stone size	0.94	0.53–1.67	0.84	–	–	–
Stone site						
Upper	Ref					
Middle	0.0	0.0–0.0	0.99	–	–	–
Lower	1.85	0.33–10.28	0.48	–	–	–
*Patients with stone size > 10 mm*						
Age	1.04	0.99–1.08	0.11	–	–	–
Hydronephrosis	0.24	0.05–1.17	0.08	–	–	–
UWT	1.91	1.37–2.65	< 0.001	3.08	1.85–5.13	< 0.001
Stone size	1.01	0.87–1.17	0.88	–	–	–
Stone site						
Upper	Ref					
Middle	0.2	0.04–1.03	0.054	0.2	0.03–1.23	0.083
Lower	1.49	0.62–3.61	0.37	8.49	2.19–32.89	0.002

*UWT* ureteral wall thickness

**Fig. 1:**
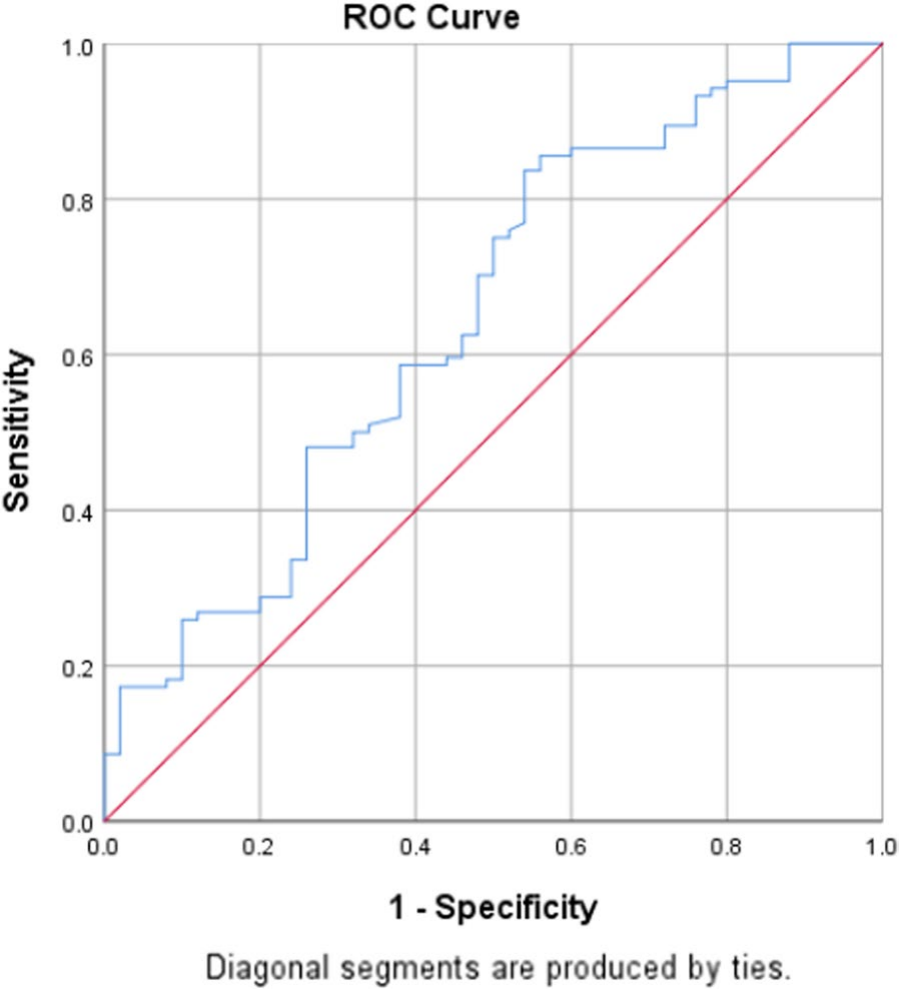
ROC curve for UWT all patients

**Fig. 2:**
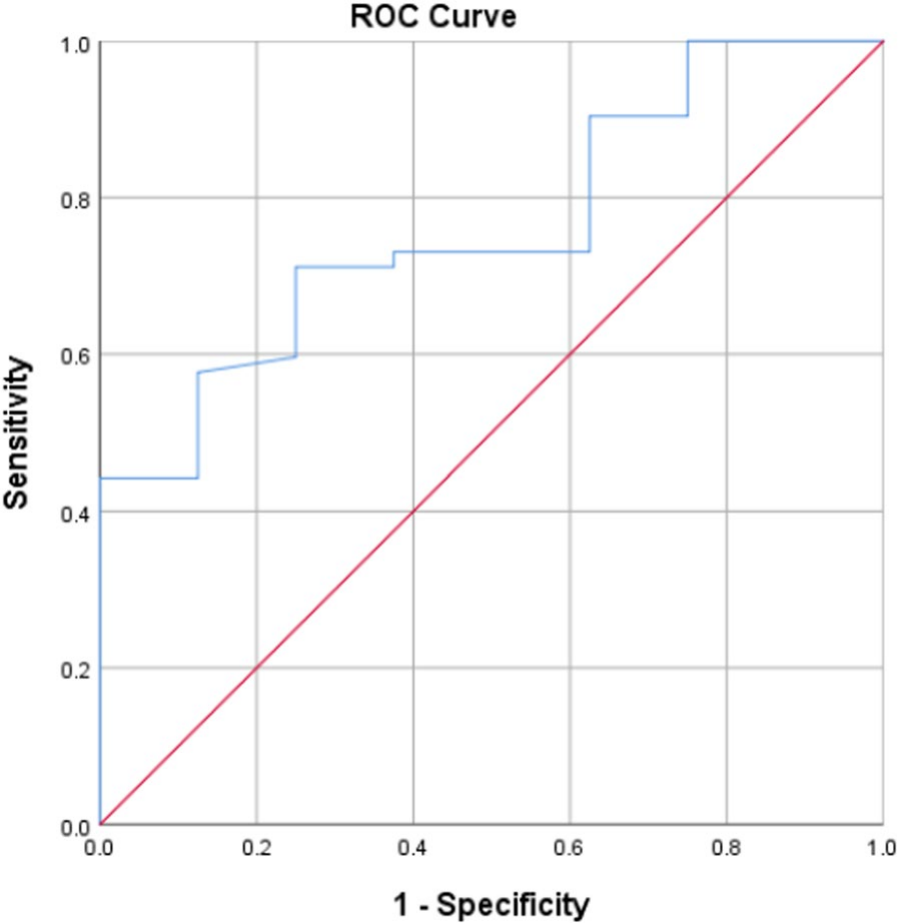
ROC curve for UWT in patients with stones ≤ 10 mm

**Fig. 3:**
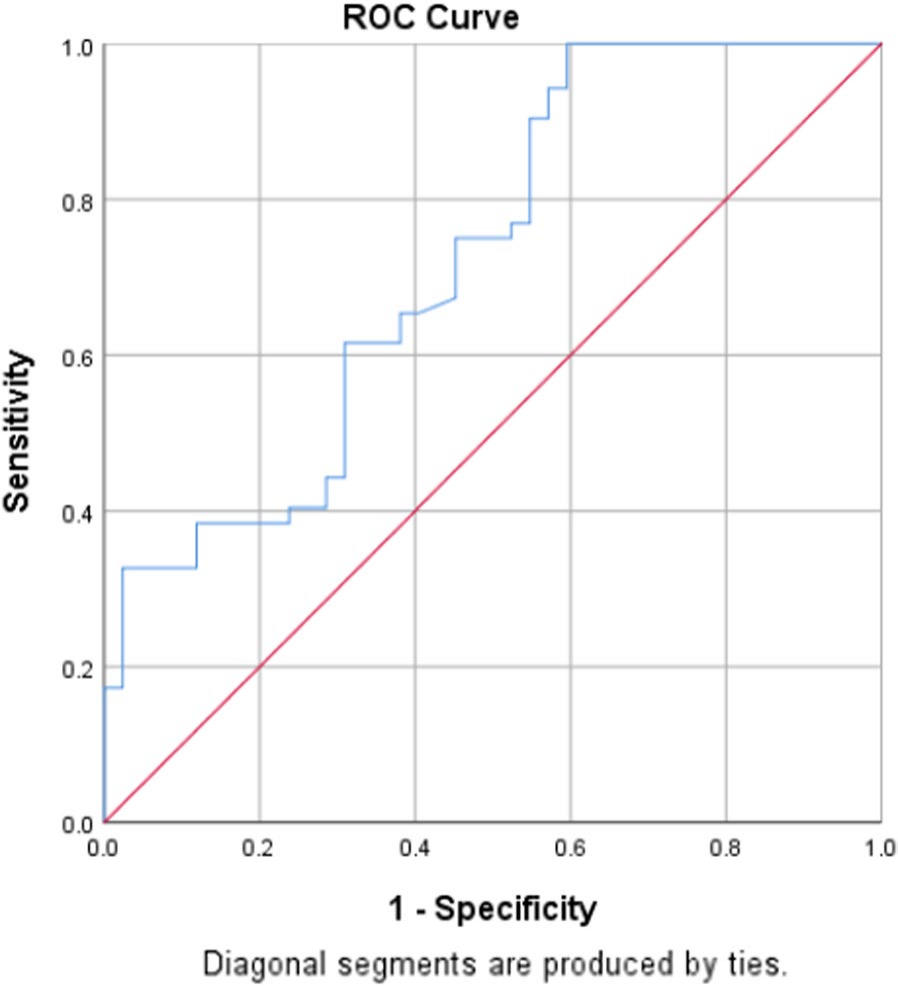
ROC curve for UWT in patients with stone size > 10 mm

In our study, the normal thickness of the ureteric wall is about 1 mm. ROC curve analysis revealed good power of UWT in discrimination of stone impaction in all patients (Fig. 1) [AUC (95% CI) 0.65 (0.55–0.74)] at a cut-off of 3.8 mm, in patients with stone size ≤ 10 mm (Fig. 2) [AUC (95% CI) 0.76 (0.61–0.91)] at a cut-off of 4.1 mm and in patients with stone size > 10 mm (Fig. 3) [AUC 95% CI] 0.72 (0.62–0.83)] at a cut-off of 3.0 mm (Table 5).

**Table 5:** Sensitivity and specificity of UWT in prediction of stone impaction

	Cut-off	AUC (95% CI)	Sensitivity	Specificity
All patients	3.8	0.65 (0.55–0.74)	75.0	50.0
Patients with stone size ≤ 10 mm	4.1	0.76 (0.61–0.91)	71.2	75.0
Patients with stone size > 10 mm	3.0	0.72 (0.62–0.83)	75.0	54.8

## Discussion

**Fig. 4:**
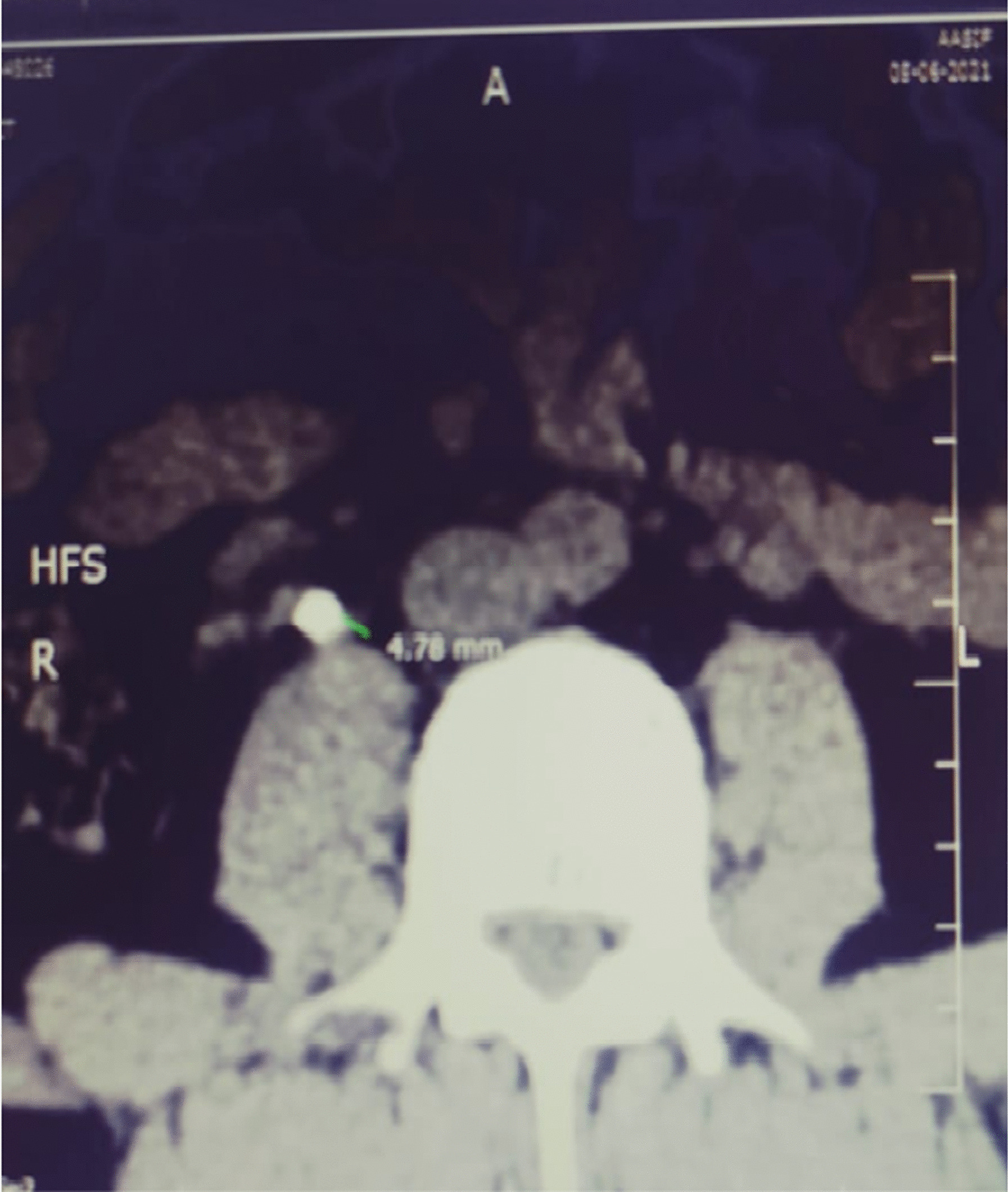
NCTUT showing uerteric wall thickness measurement in preoperative assessment

The normal thickness of the ureteric wall is about 1 mm [1]. Stone impaction causes more edema, inflammations, and hypertrophy of the ureteric wall leading to polyp formation around the stone that increases UWT at the impacted area. So, there is a correlation between stone impaction and UWT at the site of the stone (Fig. 4) [17–19].

We hypothesize that the increase of UWT on NCCT is related to edema and inflammation at the area of the stone impaction that corresponds to the endoscopic finding of ureteroscopy. The present study emphasized the clinical relevance of UWT in the preoperative prediction of ureteric stones impaction. Applying a cut-off of 3.8 mm for all patients, we found that high UWT was associated with poor endoscopic appearances related to impaction, longer operation time, and worse SFR. These findings suggest that preoperative UWT may predict the presence of impacted stones and adverse clinical outcomes in patients with ureteral stones undergoing URSL; this may change the consent process, make it less likely to offer ESWL, mean a more experienced team to do the operation and more likely to offer antegrade URS for big upper ureteric stone.

Our findings are generally in accordance with many previous reports. In the study of Yoshida et al. [12], the authors highlighted the role of UWT in the prediction of ureteral stone impaction. They suggested a UWT cut-off of 3.49 mm can be a reliable indicator of stone impaction with long operative time and less stone-free rate. In another work, it was suggested that peri-calculus ureteral thickness above, around, and below the calculus on CT are predictive of ureteral stone impaction [15].

In addition, Mishra et al. [20] found that in the majority of patients (90%) with impacted stones, the maximum UWT around the stone site was > 4.8 mm. Recently, Özbir et al. [14] integrated UWT into a formula (impacted stone formula) that comprised in addition stone size and hydronephrosis and was claimed to predict stone impaction with high sensitivity and specificity.

However, these approaches have apparent shortcomings. The study of Yoshida et al. [12] suggested a single UWT cut-off as a predictor of stone impaction regardless of the stone size. Failure to integrate stone size in this approach markedly limits its clinical value [12]. While the formula introduced by Özbir et al. [14] is impressive, its clinical applicability remains questionable, considering their study is a single-center study with a small sample size.

Instead of the mentioned approaches, the present study sought to make more clinical sense of UWT as a predictor of ureteral wall thickness. From our point of view, it’s unlogic to suggest a single UWT cut-off for the prediction of stone impaction irrespective of its size, considering the fact that there is a consensus that stone size remains the single determinant factor of stone impaction. So, we proposed to stratify the studied patients according to their stone size into those with stone size ≤ 10 mm and the others with stone size > 10 mm. Interestingly, the UWT cut-off predictive of stone impaction significantly differed between these groups and the suggested cut-off for all patients.

In the former group with stone size ≤ 10 mm, we found that stone impaction was significantly associated with higher PAPD, PUD, and UWT. In comparison, in patients with stone size > 10 mm, stone impaction was related to higher UWT, more stone number, and higher frequency of stones located in the lower ureter.

Multiple parameters were associated with stone impaction. Older age is an independent parameter for stone impaction. Previous studies reported that failure of SWL due to stone impaction is correlated with older patients [21, 22]. In contrast to our results, Yoshida et al. [12] reported a correlation between stone impaction and young age.

Previous studies reported multiple factors not depending on NCCT or endoscopic findings to predict stone impaction post-URSL [23]. Another study found that periureteral edema and rim sign at NCCT were not associated with stone impaction or SFR [24]. Our study investigated multiple predictors of stone impaction and its correlation with stone size depending on NCCT and endoscopic findings during URSL.

Yoshida et al. [12] reported that high UWT was strongly correlated with the presence of ureteral edema (n = 47, 89.3%), with 37 (78.7%) of these patients fulfilling the definition of impacted stone. Although 46 patients (55.4%) with low UWT also had ureteral edema, only 13 (15.6%) fulfilled the definition of impacted stones [12].

In our study, in the former group with stone size ≤ 10 mm, 8 (50%) with impacted stone had ureteral edema. In the group with stone size > 10 mm, 42 (72.4%) with impacted stones had ureteral edema; therefore, these results support our hypothesis and suggest that the degree of ureteral edema caused by stone impaction may be associated with the degree of UWT.

However, the current study is prospective and unique in addressing the prediction of ureteral stone impaction utilizing the preoperative ureteral wall thickness and justified by other predictive factors, including the stone size; Still, we have some limitations, including a lack of stone composition analysis and its relation to other factors as a predictor for stone impaction. The stone density in our study is low, reflecting the higher prevalence of uric acid stones among the study population, which could affect the outcomes, specifically the Lasering time. No anti-inflammatory corticosteroid drugs were taken before surgery to delay the time between the scan and the surgery.

## Conclusion

The present study concludes stratifying ureteric stones according to size would render UWT as a more practical and clinically-oriented approach for preoperative prediction of stone impaction.

## Data Availability

All data generated or analyzed during this study are included in this published article.

## References

[1] Mugiya S, Ito T, Maruyama S (2004). Endoscopic features of impacted ureteral stones. J Urol.

[2] Xi Q, Wang S, Ye Z (2009). Combined removal of stones with resection of concurrent pathologic ureter may be a preferred treatment for impacted ureteral stones with stricture lesions. J Endourol.

[3] Fam XI, Singam P, Ho CC (2015). Ureteral stricture formation after ureteroscope treatment of impacted calculi: a prospective study. Korean J Urol.

[4] Hsu JM, Chen M, Lin WC (2005). Ureteroscopic management of sepsis associated with ureteral stone impaction: is it still contraindicated?. Urol Int.

[5] Wang CJ, Hsu CS, Chen HW (2016). Percutaneous nephrostomy versus ureteroscopic management of sepsis associated with ureteral stone impaction: a randomized controlled trial. Urolithiasis.

[6] Seitz C, Tanovic E, Kikic Z (2007). Impact of stone size, location, composition, impaction, and hydronephrosis on the efficacy of holmium: YAG-laser ureterolithotripsy. Eur Urol.

[7] Elashry OM, Elgamasy AK, Sabaa MA (2008). Ureteroscopic management of lower ureteric calculi: a 15-year single-centre experience. BJU Int.

[8] Legemate JD, Wijnstok NJ, Matsuda T (2017). Characteristics and outcomes of ureteroscopic treatment in 2650 patients with impacted ureteral stones. World J Urol.

[9] Gdor Y, Gabr AH, Faerber GJ (2008). Success of laser endoureterotomy of ureteral strictures associated with ureteral stones is related to stone impaction. J Endourol.

[10] Elibol O, Safak KY, Buz A (2017). Radiological noninvasive assessment of ureteral stone impaction into the ureteric wall: a critical evaluation with objective radiological parameters. Investig Clin Urol.

[11] Tran TY, Bamberger JN, Blum KA (2019). Predicting the impacted ureteral stone with computed tomography. Urology.

[12] Yoshida T, Inoue T, Omura N (2017). Ureteral wall thickness as a preoperative indicator of impacted stones in patients with ureteral stones undergoing ureteroscopic lithotripsy. Urology.

[13] Sarica K, Eryildirim B, Akdere H (2019). could ureteral wall thickness have an impact on the operative and post-operative parameters in ureteroscopic management of proximal ureteral stones?. Actas Urol Esp.

[14] Özbir S, Can O, Atalay HA (2020). Formula for predicting the impaction of ureteral stones. Urolithiasis.

[15] Chandhoke R, Bamberger JN, Gallante B (2020). Peri-calculus ureteral thickness on computed tomography predicts stone impaction at time of surgery: a prospective study. J Endourol.

[16] White NM, Balasubramaniam T, Nayak R, Barnett AG (2022). An observational analysis of the trope “A p-value of < 0.05 was considered statistically significant” and other cut-and-paste statistical methods. PLoS ONE.

[17] Sarica K, Kafkasli A, Yazici Ö (2015). Ureteral wall thickness at the impacted ureteral stone site: a critical predictor for success rates after SWL. Urolithiasis.

[18] Deliveliotis C, Chrisofos M, Albanis S (2003). Management and follow-up of impacted ureteral stones. Urol Int.

[19] Khalil M (2013). Management of impacted proximal ureteral stone: extracorporeal shock wave lithotripsy versus ureteroscopy with holmium: YAG laser lithotripsy. Urol Ann.

[20] Mishra AK, Kumar S, Dorairajan LN (2020). Study of ureteral and renal morphometry on the outcome of ureterorenoscopic lithotripsy: the critical role of maximum ureteral wall thickness at the site of ureteral stone impaction. Urol Ann.

[21] Morgentaler A, Bridge SS, Dretler SP (1990). Management of the impacted ureteral calculus. J Urol.

[22] Sarica K, Eryildirim B, Sahin C (2016). Impaction of ureteral stones into the ureteral wall: is it possible to predict?. Urolithiasis.

[23] Imamura Y, Kawamura K, Sazuka T (2013). Development of a nomogram for predicting the stone-free rate after transurethral ureterolithotripsy using semi-rigid ureteroscope. Int J Urol.

[24] Seitz C, Memarsadeghi M, Fajkovic H (2008). Secondary signs of non-enhanced CT prior to laser ureterolithotripsy: is treatment outcome predictable?. J Endourol.

